# E2E-RDS: Efficient End-to-End Ransomware Detection System Based on Static-Based ML and Vision-Based DL Approaches

**DOI:** 10.3390/s23094467

**Published:** 2023-05-04

**Authors:** Iman Almomani, Aala Alkhayer, Walid El-Shafai

**Affiliations:** 1 Computer Science Department, King Abdullah II School for Information Technology, The University of Jordan, Amman 11942, Jordan; 2 Security Engineering Laboratory, Computer Science Department, Prince Sultan University, Riyadh 11586, Saudi Arabia or walid.elshafai@el-eng.menofia.edu.eg (W.E.-S.); 3 Department of Electronics and Electrical Communications Engineering, Faculty of Electronic Engineering, Menoufia University, Menouf 32952, Egypt

**Keywords:** ransomware, malware, cybersecurity attacks, static analysis, vision-based detection system, transfer learning, fine-tuning, machine learning, deep learning

## Abstract

Nowadays, ransomware is considered one of the most critical cyber-malware categories. In recent years various malware detection and classification approaches have been proposed to analyze and explore malicious software precisely. Malware originators implement innovative techniques to bypass existing security solutions. This paper introduces an efficient End-to-End Ransomware Detection System (E2E-RDS) that comprehensively utilizes existing Ransomware Detection (RD) approaches. E2E-RDS considers reverse engineering the ransomware code to parse its features and extract the important ones for prediction purposes, as in the case of static-based RD. Moreover, E2E-RDS can keep the ransomware in its executable format, convert it to an image, and then analyze it, as in the case of vision-based RD. In the static-based RD approach, the extracted features are forwarded to eight various ML models to test their detection efficiency. In the vision-based RD approach, the binary executable files of the benign and ransomware apps are converted into a 2D visual (color and gray) images. Then, these images are forwarded to 19 different Convolutional Neural Network (CNN) models while exploiting the substantial advantages of Fine-Tuning (FT) and Transfer Learning (TL) processes to differentiate ransomware apps from benign apps. The main benefit of the vision-based approach is that it can efficiently detect and identify ransomware with high accuracy without using data augmentation or complicated feature extraction processes. Extensive simulations and performance analyses using various evaluation metrics for the proposed E2E-RDS were investigated using a newly collected balanced dataset that composes 500 benign and 500 ransomware apps. The obtained outcomes demonstrate that the static-based RD approach using the AB (Ada Boost) model achieved high classification accuracy compared to other examined ML models, which reached 97%. While the vision-based RD approach achieved high classification accuracy, reaching 99.5% for the FT ResNet50 CNN model. It is declared that the vision-based RD approach is more cost-effective, powerful, and efficient in detecting ransomware than the static-based RD approach by avoiding feature engineering processes. Overall, E2E-RDS is a versatile solution for end-to-end ransomware detection that has proven its high efficiency from computational and accuracy perspectives, making it a promising solution for real-time ransomware detection in various systems.

## 1. Introduction

One of the most prevalent types of malicious software is ransomware [1,2]. Ransomware hijacks the user’s device by locking the device or encrypting the user’s data, requesting monetary payment in exchange for the locked resources, often causing irreversible information losses and enduring high economic costs. According to [3], ransomware threat attacks surpassed all other types of cybersecurity threats with around 304.7 million attacks in the first half of 2022. Therefore, there is an urgent need to develop ransomware detection systems to face the growing scourge of ransomware applications. Ransomware detection systems identify whether an application is ransomware or benign. Presently, most anti-virus software implements the signature-based approach [4]. Even though signature-based schemes consume fewer resources and less time, they are vulnerable to zero-day attacks. Additionally, the signature-based systems lack the ability to identify ransomware applications that utilize obfuscation and polymorphism techniques. Furthermore, the presence of ransomware development tool kits such as RaaS (Ransomware as a Service) contributes to the growing number of ransomware attacks [5].

To address the aforementioned issues, Machine Learning (ML) techniques are utilized in implementing ransomware analysis schemes [1,2,6,7,8]. In ransomware analysis, the functionality of a certain application is determined to classify ransomware and benign apps. The process of ransomware classification generally falls into three categories: static analysis, dynamic analysis, and vision-based analysis. In static analysis, the ransomware APK is reverse-engineered in order to retrieve the source code, strings, and resources of the application [9]. This approach lacks the ability to monitor the suspicious behavior of the ransomware since it does not involve an APK execution process. Consequently, the source code of the ransomware might bypass the static analysis even though it contains harmful methods that are invoked only during the run time process. Dynamic analysis detects ransomware applications by executing their APKs in order to observe their run-time behaviors. As a result, several features can be investigated through the run-time analysis, such as network traffic, CPU and memory usage, and system calls [10]. Dynamic analysis is typically performed in a controlled virtual environment, such as emulators, to avoid harming real devices, which might affect ransomware behavior.

In the vision-based RD analysis, the Android apps are converted to visual images before in-depth training and testing mechanisms using the developed deep learning (DL) classifiers [11,12,13,14,15]. The main improvements of vision-based analysis compared to other static-based or dynamic-based RD detectors are that they lessen the computational cost and avert reverse engineering steps needed in static-based RD analysis [16]. Moreover, they do not require to operate particular and isolated running ecosystems to check the behavior of the ransomware apps as required in dynamic-based RD analysis [17].

The research problem addressed in this paper is the need for an effective ransomware detection system to identify and prevent attacks on Android mobile systems. So, we proposed an end-to-end ransomware detection system that used a combination of machine learning and deep learning approaches based on static-based and vision-based analyses to detect ransomware attacks. Thus, this research aims to develop a system that can detect ransomware attacks in real-time on Android operating systems.

**Figure 1:**
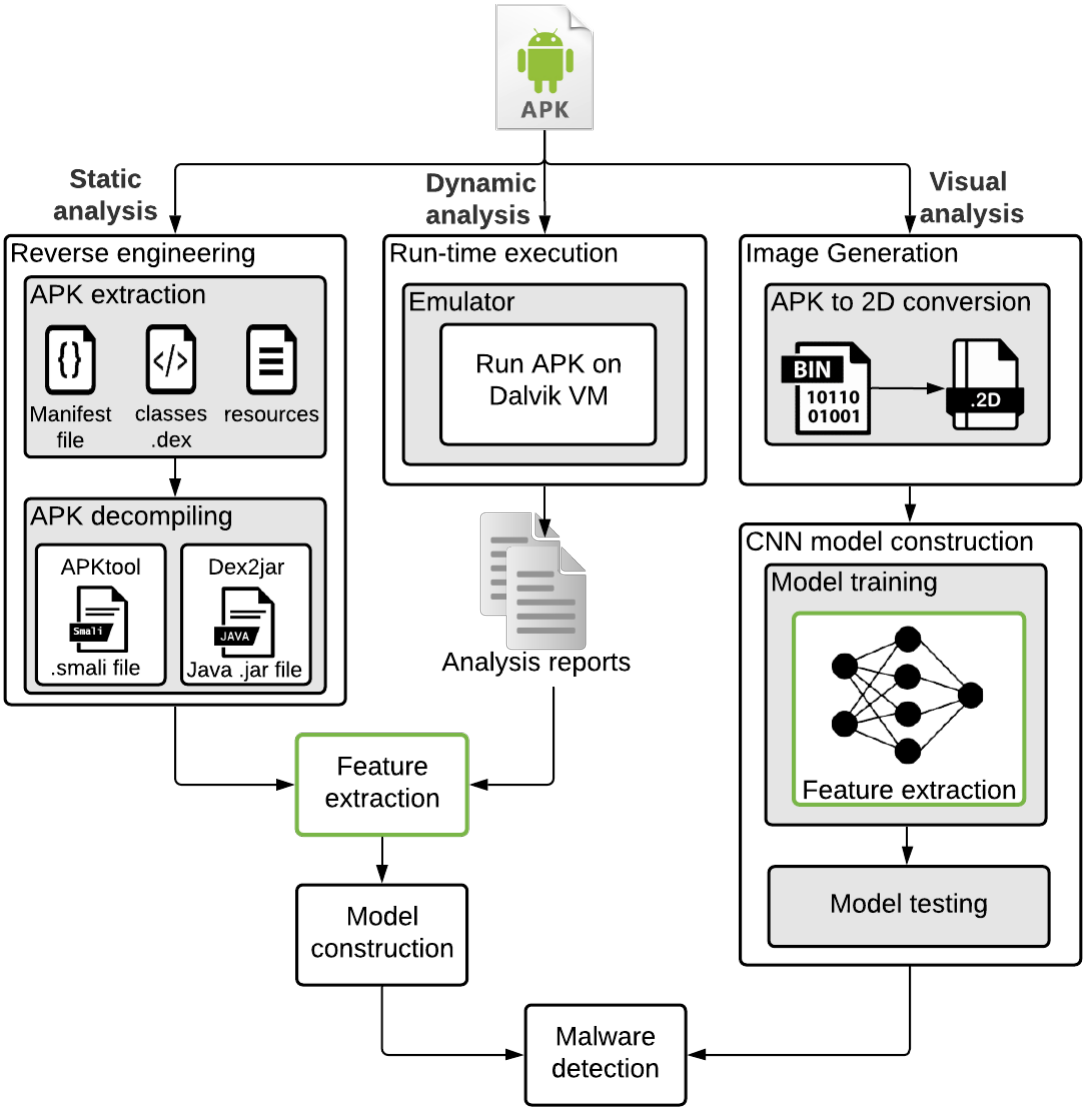
Malware classification approaches.

Consequently, the research problem behind this work is the growing threat of ransomware attacks on mobile systems and the need for an effective and efficient ransomware detection system. This motivated us to propose a system combining static-based machine learning and vision-based deep learning approaches to detect ransomware attacks. This is because most existing related works apply only one of these approaches in their solutions. They did not try to benefit from the advantages of both approaches within the same context. As a result, we developed a system that can identify ransomware attacks accurately and quickly, allowing for timely responses and reducing the impact of such attacks on organizations and individuals. Consequently, the static and dynamic analyses are built on feature engineering in which a database of malware features is deployed to implement the machine learning classification scheme. The rapid development of malware with constant changes raises the challenge of manually updating the malware feature database. Therefore, to reduce the cost of malware feature engineering, visual-based approaches are utilized to develop malware detection systems. In visual-based systems such as convolutional neural networks (CNN), the feature engineering process is embedded within the model construction phase, as shown in Figure 1.

The conventional detection techniques might not be efficient enough to detect new malware families, including ransomware, without causing high computational processing and memory overheads. Additionally, the traditional malware detection techniques disclose detection performance degradation due to a limited number of malware samples. Consequently, the key contributions of this paper are summarized as follows:

- Proposing an efficient, comprehensive end-to-end RD system (E2E-RDS) that composes different static-based ML and vision-based DL classifiers.
- Developing eight different ML models and 19 different DL models and exhaustively checking their ransomware detection capabilities.
- Examining the proposed E2E-RDS classification performance using static and vision-based RD approaches.
- Testing the proposed vision-based RD approach’s performance using different color and grayscale visual data of the ransomware and begin apps.
- Assessing the proposed E2E-RDS’s performance using various detection and security evaluation parameters.

The rest of this paper proceeds as follows. Section 2 summarizes the most recently published and relevant static-based, dynamic-based, and vision-based RD systems in the literature studies. Section 3 explains the proposed end-to-end RD system. Section 4 offers the obtained simulation results and comparisons. Section 5 gives the concluding remarks and some future suggestions.

## 2. Literature Survey

The advent of Android ransomware applications that threaten security measures has motivated several analysis approaches for ransomware classification [18]. This section presents an overview of ransomware detection techniques used in related studies. Table 1 presents and analyzes the recent and relevant RD systems published in the literature studies. So, Table 1 compares selected papers published in 2020 and after. This range witnessed the flourishing of visual-based malware detection. Therefore, this literature survey mainly discusses three approaches recently utilized in Android ransomware detection, which are the static-based, dynamic-based, and visual-based approaches. Table 1 summarizes the comparative analysis among these approaches in terms of their aim, the dataset used, the approach followed, the ML/DL models utilized, the system input, features considered, and their solution performance concerning accuracy.

### 2.1. Static-Based Analysis

Static analysis efficiently implements ransomware detection systems without executing the malware code. Alkhayer et al. proposed a static analysis framework in which the decompiled APK is parsed in order to build a data set of the applications features [9,19]. Initially, the decompilation process of the APKs retrieves the Manifest file and the .smali files. After that, these files are scanned via a parsing tool, ASParse, resulting in the creation of a feature dataset. In [20], a permission-based detection system was developed to determine Android permissions that contribute to identifying ransomware with high accuracy. Zhang et al. constructed a static framework that implements a self-attention detection approach based on opcode sequence in order to classify ransomware [21].

Almomani et al. investigated the most used features of the latest version of Android (version 11) by ransomware [1]. The parsed features were utilized in building a ransomware detection system by implementing Random Forest (RF), Naive Bayes (NB) Sequential Minimal Optimization (SMO), and Decision Tree (DT). In [2], the authors simulated the real-word by implementing a hybrid evolutionary technique utilizing a highly imbalanced dataset. The dataset considered by this research consists of 10,153 Android apps, where only about 5% of the collected applications were ransomware.

In [22], an improved framework named for zero-day ransomware detection was proposed. The framework employed deep learning-based unsupervised feature extraction to extract features from the input data and a cost-sensitive Pareto Ensemble classifier to classify the data into either ransomware or benign. The cost-sensitive approach assigned different costs to misclassification errors to address the issue of imbalanced data distribution. The proposed framework showed better performance in detecting zero-day ransomware compared to traditional classifiers, highlighting the effectiveness of the approach.

**Table 1:** Summary of existing malware/ransomware detection schemes.

Work	Aim	Dataset	Approach	Models	System Input	Features	Accuracy
[10]	To extract system calls by developing a dynamic analysis. The extracted class were fed to different ML algorithms.	400 B, 400 R	Dynamic	Random Forest, J48, Naïve Bayes	APK	system calls	98.31%
[9]	To propose a static analysis in which the decompiled APK is parsed in order to build a data set of the application’s features.	-	Static	-	Decompiled APK	Permissions, API calls	-
[20]	To determine Android permissions that contribute to identifying ransomware with high accuracy.	500 B, 500 M	Static	RF, J48, SMO, Naive Bayes	Decompiled APK	Permissions	96.9%
[23]	To implement malware classification on a multi-class level by combining static feature extraction and deep learning.	200 KB, 200 KM	Visual	Scratch model	Decompiled APK	set of stactic features	93.36%
[24]	To dynamically analyze an application by intercepting its network traffic.	62 B, 130 M	Dynamic	string comparison	APK	System calls	92%
[25]	To develop a ransomware detection system by observing the behavior of system calls.	502 B, 500 M	Dynamic	-	APK	System calls	98.6%
[21]	To develop a self-attention detection system based on opcode sequence.	100 B, 1787 M	Static, DL	N-gram opcodes	APK	Opcode sequence	89.5%
[26]	To develop a hybrid-based ransomware detection system that combines both static and dynamic analysis.	500 B, 500 M	Hybrid (static, dynamic)	-	APK	API calls	-
[1]	To investigate the most used features by ransomware and utilize them in building a ransomware detection system.	501 B, 500 M	Static	RF, DT, SMO, NB	APK	Permissions, API calls	98.3%
[2]	To classify ransomware utilizing an evolutionary approach by deploying the SVM algorithm.	9653 B, 500 M	Static	particle swarm optimization algorithm, SVM, SMOTE	APK	Permissions, API calls	-
[27]	To improve the malware detection accuracy by deploying fine-tuned CNN models.	9341 M	CNN	nine different models	Gray-scale image of APK	-	99.97%
[28]	To analyze the local binary pattern by detecting the irregularity of the image texture enabling immediate detection before execution.	500 B, 305 M	CNN	local binary pattern (LBP)-based technique, SVM	Gray-scale image of APK	-	87.9%
[29]	To effectively classify packed and unpacked malicious software by deploying an ensemble-based CNN scheme.	9339 M	CNN	VGG16, ResNet-50	Gray-scale image of APK	-	99%
[30]	To investigate the effectiveness of the visual-based approach by employing twelve different CNN models on a large dataset.	12,971 B 20,199 M	CNN	VGG3, ResNet-50	Gray-scale, colored, Markov, Gabor Markov images	-	99.97%
[31]	To construct a Markov image that contains the malware statistics by deploying bytes transfer matrices.	4020 M	CNN	Scratch model	Markov image of .dex file	-	97.364%

In [32], the authors proposed a new approach named spline interpolation-envisioned neural network-based for detecting ransomware. This approach combined spline interpolation and neural network techniques to improve accuracy and security. The simulation results proved the security of the proposed approach against various possible attacks. In [33], a feature selection-based ransomware detection model is presented. This model utilized information gain and a genetic algorithm to generate feature sets that can be used with any machine learning classifier. Different evaluation experiments were conducted on a real-world dataset, demonstrating the feasibility of the proposed model for ransomware detection.

In [34], the authors proposed a new optimized ML method for ransomware detection in an Internet of Things (IoT) environment. This method used the dwarf mongoose optimization algorithm to optimize the selection of parameters in an extreme ML classifier. The proposed method was evaluated on a benchmark dataset and outperformed other state-of-the-art methods in terms of accuracy and speed.

### 2.2. Dynamic-Based Analysis

An example of the dynamic analysis scheme is ShadowDroid which is a system that dynamically analyzes an application by intercepting its network traffic [24]. To collect the needed data, ShadowDroid set up a VPN on the targeted device and performed a string-matching algorithm to detect private information. The proposed system aims to enable users to take the correct response strategy. In [26], a hybrid approach was followed in which both static and dynamic analyses were implemented.

Abdullah et al. developed an Android ransomware classification system using dynamic-based analysis [10]. The proposed system executed the ransomware application on a virtual environment to extract the system calls. Subsequently, the extracted system calls were fed into three different machine learning algorithms, Random Forest, Naïve Bayes, and J48. Random Forest exceeded the other algorithms by achieving an accuracy of 98.31%. Furthermore, Wen et al. built a ransomware detection system based on observing system calls generated by the ransomware behaviors [25]. The proposed system focused only on the encryption-type ransomware family.

In [35], the authors presented a comprehensive analysis of techniques to evade ransomware detection techniques that use behavioral classifiers. These techniques can evade behavioral features commonly used by classifiers to detect malware, including features that are difficult to disguise and are intrinsically related to the behavior of malware processes. The authors evaluated the effectiveness of these techniques against state-of-the-art ransomware detection methods and showed that the proposed evasion techniques could effectively evade behavioral classifiers.

In [36], the authors proposed a dynamic feature dataset for detecting ransomware using machine learning algorithms. The dataset included features related to classification, encryptor, locker, and other relevant factors. The authors evaluated the dataset using three machine learning algorithms: gradient-boosted regression trees, random forest, and neural networks. They found that all three achieved an average accuracy of over 0.98 using a 10-fold cross-evaluation.

### 2.3. Visual-Based Analysis

Recently, deep learning has arisen as a distinguished malware classification approach. However, there is a shortage of research on detecting ransomware deploying a visual-based approach [28,37,38]. Hence, general malware texture-based classification systems were reviewed. Sharma et al. analyzed local binary patterns by detecting the irregularity of the image texture, enabling immediate detection before execution [28]. Consequently, the proposed system exposes any malicious operations injected into the ransomware byte code.

In some research works, pre-trained models can be utilized by implementing a fine-tuning phase in which the knowledge of these models is transferred. For example, El-Shafai et al. deployed fine-tuned CNN models to improve the malware detection accuracy without constructing training models [27,39]. The visual-based approach might be combined with the static approach in order to implement a multi-class classification of malware applications. In [23], the authors have proposed DIDroid, which converts the static features into 2D images aiming to perform malware characterization on a multi-class level. At first, the APKs were decompiled to extract the required features. Hence, the extracted features were converted into 2D images and fed to the deep learning model.

To effectively classify packed and unpacked malicious software, the authors of [29] deployed VGG16 and ResNet-50 CNN models utilizing the Malimg dataset to develop the proposed detection system. Pinhero et al. investigated the effectiveness of the vision-based approach by employing twelve different CNN architectures by modifying the VGG3 and ResNet-50 models [30]. A further step towards utilizing the vision-based approach in implementing malware classification schemes on the byte level was proposed by [31]. In [31], the authors constructed a Markov image containing malware statistics by deploying bytes transfer matrices.

In [40], a method for evading ransomware detection technologies that use entropy measurements to identify encrypted data was proposed. This method involved the application of various encoding algorithms, including base64 and different file formats, to alter the randomness of the data and make it appear less random, thereby bypassing the entropy-based detection methods used by ransomware detection technologies. While the proposed method was found to be effective against several ransomware detection tools, the authors caution that it may not be effective against all detection technologies, and further research is needed to develop more robust detection techniques. In [41], the authors presented a new method for detecting ransomware using graph embedding to represent the portable executable header. The proposed approach overcame the limitations of traditional signature-based and machine learning-based methods that require large amounts of labeled data. The authors evaluated their method on a dataset of over 2000 ransomware and non-ransomware samples and found that it achieved high accuracy and outperformed existing methods.

As can be observed from Table 1, no existing study has considered both static/dynamic and vision-based approaches within the same context to examine their performance in detecting malware in general and ransomware in specific. Consequently, this has motivated this research to conduct a deep study on the impact of applying both approaches while considering the same malware datasets and implementation environments.

## 3. Proposed End-to-End Ransomware Detection System

This section discusses the details of the proposed end-to-end ransomware detection system. End-to-end means full utilization of different types of analysis methods, whether the ones that depend on reverse engineering where the malware code is recovered and parsed and several features are gathered, such as the case in the static-based analysis or by taking the malware executable code as is (PE: Portable Executable), converting it to an image, and then applying analysis as in the case of vision-based systems. The aim is to investigate the advantage of each one of them to ensure efficient detection of ransomware apps using the same inputs and environments.

**Figure 2:**
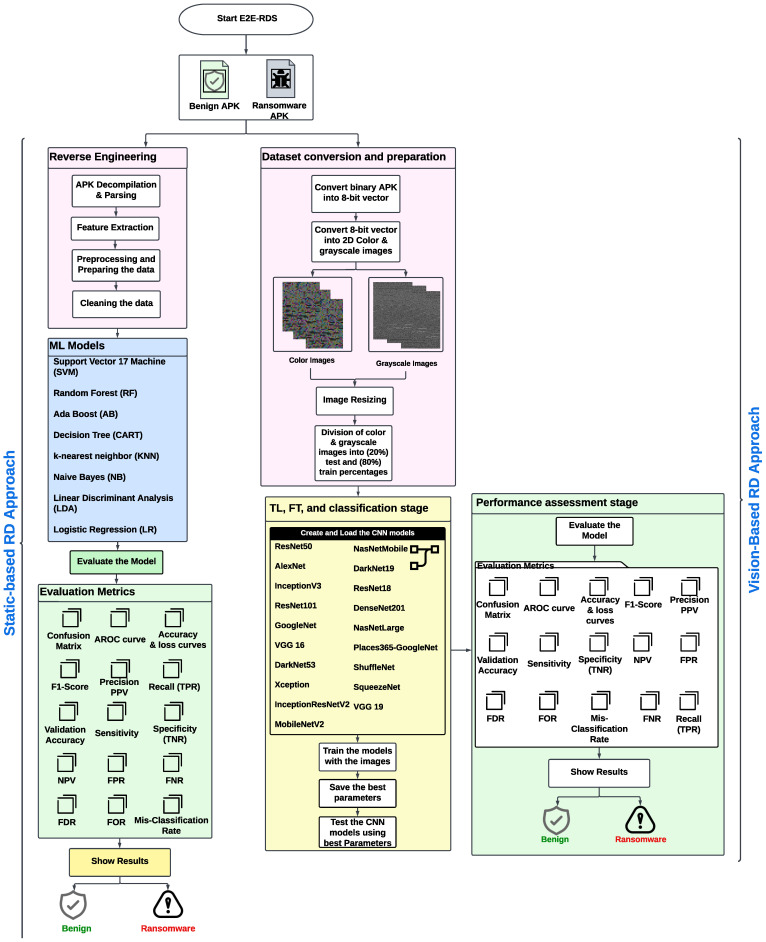
Structure of the proposed end-to-end ransomware detection system.

Figure 2 shows the high-level diagram of the E2D-RDS system that composes two proposed approaches to efficiently detect ransomware apps. The first static-based RD approach has utilized ML models, as will be discussed in Section 3.1. In contrast, the second vision-based RD approach has used different CNN models by exploiting the substantial advantages of FT and TL mechanisms, as will be clarified in Section 3.2.

Here are the main steps of the proposed E2E-RDS system that are presented in Figure 2:

- Load dataset containing ransomware and non-ransomware files.
- Preprocess the dataset by extracting features.
- Split extracted features dataset into training and testing sets.
- Train static-based machine learning models on the training set.
- Test the machine learning models on the testing set.
- Convert the dataset samples into color and gray images.
- Split images dataset into training and testing sets.
- Train vision-based deep learning models on the training set.
- Test the deep learning models on the testing set.
- Check the output of the static-based and vision-based models.
- If the output indicates ransomware, flag the file as malicious.
- If the output indicates non-ransomware, flag the file as safe.
- Evaluate the performance of the system using different relevant metrics such as accuracy, precision, recall, and other detection metrics.
- Fine-tune the models and retrain if necessary.
- Save the final models for use in future detections.

### 3.1. The Proposed Static-Based RD Approach

As indicated in Figure 2, in the static-based RD approach, the binary portable APK files are first decompiled to extract the AndroidManifest.xml binary file, which comprises the APK’s metadata and all well-defined permissions by the Android APKs. In the decompile process, we used the APKtool (https://ibotpeaches.github.io/Apktool/, accessed on 1 January 2020) to decompile the zipped Android app to the manifest and SMALI files. The SMALI file signifies a well-defined class in the original binary code of the Android APK. After that, the required features of the Android APKs are obtained by analyzing the obtained SMALI and manifest files.

In the parsing process, the obtained feature set used in the proposed static-based RD approach includes 161 permissions, 228 API packages, and 389 features. The feature occurrences have been calculated using the assembled APKs by obtaining the features from the manifest and SMALI files. Therefore, all Android APKs are scanned using separate stages during the parsing process. In the first stage, the manifest file of each Android APK is parsed to count the specified features, such as permissions. Then, in the second stage, the SMALI files of each Android APK are parsed to count the utilized API packages. At the end, the total parsed extracted features are deposited in the database.

In addition, in the proposed static-based RD approach, we apply pre-processing, preparing, and cleaning mechanisms to the extracted features to remove the zero-values or null attributes and represent the extracted features in the proper format before forwarding them to the utilized ML classifiers. This approach uses eight different ML models to train and test the extracted features from the APK files. These ML classifiers are Support Vector Machine (SVM), Random Forest (RF), Ada Boost (AB), Decision Tree (CART), K-Nearest Neighbor (KNN), Naive Bayes (NB), Linear Discriminant Analysis (LDA), and Logistic Regression (LR) [42,43,44]. Finally, the detection efficacy of the used ML classifiers is evaluated using different assessment tools such as precision, recall, F1-score, ROC curve, confusion matrix, and accuracy [45,46].

### 3.2. The Proposed Vision-Based RD Approach

The efficient Ransomware Detection (RD) process is an obligatory aspect of cybersecurity applications because ransomware is a highly harmful malicious software that can infect and encrypt the users’ or organizations’ files and request payment to decipher the encrypted files. Most of the RD approaches developed recently have introduced different detection mechanisms to identify ransomware after extracting its main texture features (static-based RD) or starting its execution (dynamic-based RD). However, unfortunately, the static-based RD approaches require additional processing stages to analyze and extract the ransomware features. In addition, until recently once the ransomware app is executed and begins its attack, no currently developed dynamic-based RD approaches have been suggested that can prevent and stop its harm. Therefore, instead of using conventional static- or dynamic-based RD approaches, this section introduces a vision-based RD approach by exploiting 19 different FT CNN models. These models can inexpensively identify ransomware apps with low computation requirements and high detection performance.

In the proposed vision-based RD approach, the binary executable files of the benign and ransomware apps are converted into 2D visual (color or grayscale) images. Then, these images have been forwarded to the employed FT CNN models for a binary classification purpose to differentiate ransomware apps from benign apps. The main benefit of this approach is that it can efficiently detect and identify ransomware without using data augmentation or complicated feature extraction processes. Therefore, the FT (Fine-Tuning) and TL (Transfer Learning) techniques were only exploited in the proposed vision-based RD approach to achieve high detection accuracy. The TL process offers effective and promising solutions through the knowledge transfer of pre-trained CNN models. While in the FT technique, the pre-trained CNN layers are progressively trained by modifying their weights and the learned hyperparameters until they achieve a remarkable classification performance.

As indicated in Figure 2, the primary strategy of the proposed vision-based RD approach involves three main stages: (1) dataset conversion and preparation stage, (2) TL, FT, and classification stage, and (3) performance assessment stage. The explanations of these stages are as follows:

#### 3.2.1. Dataset Conversion and Preparation Stage

In this stage, the benign and ransomware apps of our newly collected balanced 500/500 (ransomware/benign) dataset are directly converted to both color and gray images without using any decryption, decompression, and disassembly methods. The primary goal of transforming the PE (portable executable) APKs to images is to acquire the main texture features of the benign and ransomware apps. In the conversion process, the benign and ransomware APKs are first transformed into 1D 8-bit binary vectors, and after that, these binary vectors are transformed into visual 2D color or gray images. The CNN models utilized in the proposed vision-based RD approach for the binary classification task exploited the resulting texture features extracted from the obtained images in detecting ransomware apps efficiently. Thus, the significant benefit of the proposed vision-based RD approach is that it does not need any feature extraction or reverse-engineering tasks to be performed.

**Figure 3:**
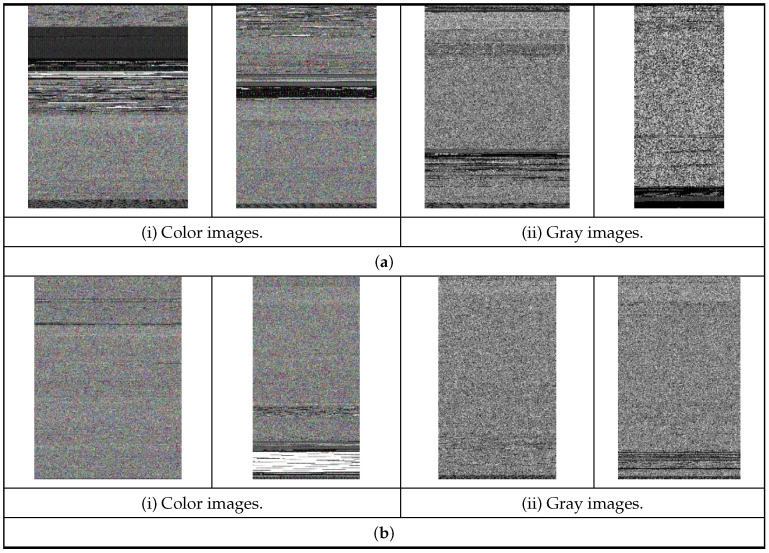
Samples of visual color and gray images of ransomware and benign apps. (**a**) Visual images of ransomware apps. (**b**) Visual images of benign apps.

Figure 3 demonstrates some visual color and gray images of the ransomware and benign apps after reorganizing the 1D binary vectors into 2D graphical arrangements. It is noticed from Figure 3 that the texture features and stripes of the obtained ransomware images are entirely different from the texture features and stripes of the benign images, either in the color or gray images. Additionally, as observed, each converted image has a different width than another image because each benign or ransomware app has a different size. So, the obtained image width is based on the size of the benign and ransomware APKs. Table 2 shows the typical different image widths of benign and ransomware APKs based on their sizes. Consequently, such remarks have inspired us to optimize and exploit the common pre-trained CNN architectures applied for image recognition and classification tasks into vision-based RD tasks.

Before forwarding the accumulated visual images of the ransomware and benign apps to the suggested fine-tuned CNN models for detection and classification purposes, these images are divided into 80% to train the layers of CNN models and 20% for testing the CNN models. These testing and training percentages of benign and ransomware images are selected randomly for the accumulated visual image datasets (color and grayscale). Additionally, as a preparation step, the obtained visual images of the benign and ransomware apps must be resized before performing the training and classification processes using a specified CNN model. This is because each one of the suggested fine-tuned CNN models has its standard size for the input visual images, as demonstrated in Table 3.

#### 3.2.2. TL, FT, and Classification Stage

As shown in Figure 2, the proposed vision-based RD approach comprises 19 various CNN models, which are ResNet50, AlexNet, InceptionV3, ResNet101, GoogleNet, VGG16, DarkNet53, Xception, InceptionResNetV2, MobileNetV2, NasNetMobile, DarkNet19, ResNet18, DenseNet201, NasNetLarge, Places365-GoogleNet, ShuffleNet, SqueezeNet, and VGG19 [47,48,49]. These models do not employ reverse engineering to extract the main features of the visual images used in the binary classification process of detecting and recognizing ransomware apps. Thus, in the proposed vision-based RD approach, we exploit the transfer learned features of the fine-tuned and optimized CNN versions of these pre-trained CNN models to detect ransomware attacks without the need to design deep CNN models learned from scratch.

The CNN models used in the proposed vision-based RD approach can be classified into two categories: (1) single-path designs and (2) multi-path designs. In the first category of single-path designs, the CNN layers are arranged in a series path with a sequential structure, and they have single input and output layers. In the second category of multi-path designs, there are multiple parallel paths for the composed CNN layers in the utilized CNN model, and thus they have multiple input and output layers. Examples of single-path CNN designs are AlexNet, VGG16, DarkNet19, and VGG19 models, wheres examples for the multi-path CNN designs are ResNet50, InceptionV3, ResNet101, GoogleNet, DarkNet53, Xception, InceptionResNetV2, MobileNetV2, NasNetMobile, ResNet18, DenseNet201, NasNetLarge, Places365-GoogleNet, ShuffleNet, and SqueezeNet models [47,48,49]. From a complexity perspective, the multi-path designs-based CNN models are more complex than the single-path-design-based CNN models. They have an additional advantage in attaining high detection accuracy and a low misclassification rate. This is due to the extensive extracted features from the input visual ransomware and benign images by different composed CNN layers during the training process.

The TL is considered a supervised learning process that trains a CNN model on one classification problem with a specific image dataset. After that, it utilizes the learned features in another and different classification challenge on a different image dataset. The significant profit of using the TL process is to decrease the examined CNN model’s training time and achieve low generalization and classification errors, thus avoiding the overfitting occurrence. In addition, the importance of employing the TL process is highlighted when the dataset used in the classification task has few samples of visual images, as in our proposed vision-based RD approach (500 ransomware samples and 500 benign samples).

Amongst the nineteen different tested CNN structures utilized in the proposed vision-based RD approach, the optimized version of the ResNet50 CNN structure attains the best detection efficiency and classification accuracy. Therefore, in our work, in-detail discussions and explanations of this CNN model (FT ResNet50) are presented. The general details and basic descriptions of the other 18 different CNN structures (AlexNet, InceptionV3, ResNet101, GoogleNet, VGG16, DarkNet53, Xception, InceptionResNetV2, MobileNetV2, NasNetMobile, DarkNet19, ResNet18, DenseNet201, NasNetLarge, Places365-GoogleNet, ShuffleNet, SqueezeNet, and VGG19) used in the proposed vision-based RD approach could be explored in [50,51,52,53,54,55,56].

**Figure 4:**
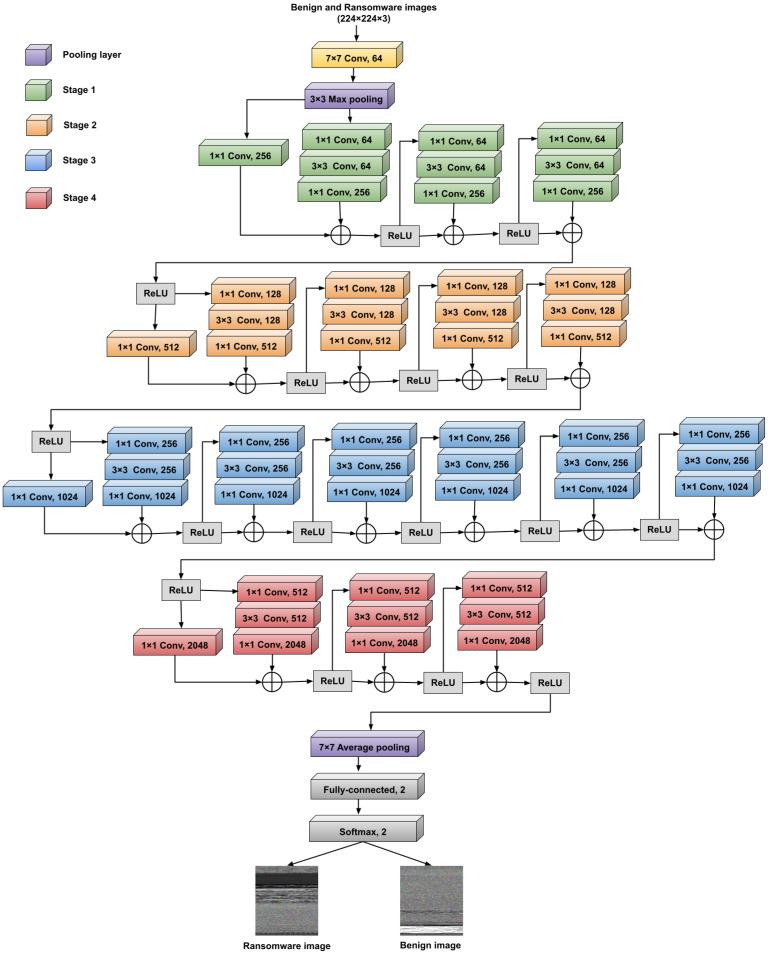
Structure of the fine-tuned ResNet50 model.

The structural design of the employed FT ResNet50 CNN model employed in the proposed RD approach is depicted in Figure 4. The pre-trained version of this ResNet50 CNN model is trained on more than ten million natural images with more than 1000 various classes composed in the common ImageNet dataset [57]. Thus, in the proposed vision-based RD approach, we exploited the resulting learned features and performed fine-tuning for the convolutional layers’ hyperparameters and weights to detect ransomware attacks efficiently and rapidly.

The utilized residual network (ResNet50) has a simpler multi-path design structure than other multi-path CNN structures. It consists of four different sequential stages of convolutional (Conv) layers with different sizes and numbers of integrated filters, as shown in Figure 4. These Conv layers extract the main features from the input visual ransomware and benign images. Each of these Conv layers in the ResNet50 structure has a batch normalization layer and a Rectified Linear Unit (ReLU) activation function that is not indicated in Figure 4 for presentation simplicity. So, there is a hidden ReLU function after each hidden batch normalization layer for each Conv layer in Figure 4. The ReLU functions are used to activate the nonlinear batch normalization layers for quickly accomplishing convergence performance and thus accelerating the training mechanism. Therefore, the ResNet50 model is faster and more accurate than other multi-path CNN structures. In addition, it can extract more features and characteristics from the input visual images of the ransomware and benign apps.

Furthermore, the ResNet50 model includes a max-pooling layer with a kernel size of 3 × 3 at the input level, and it incorporates an average pooling layer with a kernel size of 7 × 7 at the output level. The max-pooling layer determines the maximum value within the patches of the extracted feature maps. In contrast, the average pooling layer is employed to determine the average value within the patches of the extracted feature maps. Finally, the extracted features resulting from the Conv layers are forwarded to the classification layers (fully connected and softmax layers) to differentiate the ransomware samples from the benign samples. In the FT ResNet50 model, the last fully connected layer is modified to have two outputs (ransomware and benign) instead of the 1000 outputs as in the original pre-trained ResNet50 CNN model.

The main advantages of the employed FT ResNet50 model are (1) the utilization of the batch normalization layer that optimizes the parameters of the input layer to improve the CNN model performance, and thus the covariate shift is alleviated, (2) the utilization of the identity connections that protect the CNN model structure from diminishing gradient problems, (3) the utilization of residual bottleneck block designs that enhance the CNN model performance, and (4) the utilization of different sizes of kernels within the Conv layers, and thus the achievement of deep discovering and learning of the foremost characteristics and texture features from the input visual images.

As discussed in the proposed vision-based RD approach, the fine-tuning for the hyperparameters of the layers included in the employed CNN models is carried out besides exploiting TL benefits. The fine-tuning method is preferable during the training of CNN models compared to other tunning methods (e.g., shallow tuning and deep tuning, because the FT achieves higher detection performance and lower computations than shallow and deep tuning approaches). Thus, this motivates us to use the FT process in the proposed vision-based RD approach to optimize the weights and parameters of the CNN layers until achieving optimum classification results for efficiently detecting the ransomware attacks in android mobile operating systems. The optimum fine-tuning and training parameters utilized in the second proposed approach are summarized in Table 4. These optimization parameters are thoughtfully selected after performing different simulation experiments on the examined CNN models using the created visual android dataset until optimizing the testing and training operations of the employed CNN models to avoid overfitting problems.

#### 3.2.3. Performance Assessment Stage

Various detection assessment metrics have been exhaustively introduced in the literature to offer comprehensive evaluations of detection and classification algorithms [45,46]. In our performance analysis of the employed CNN models, we used various detection metrics instead of only evaluating the detection accuracy, since the utilization of only detection accuracy as an assessment metric of the classification performance is not precise from conceptual and practical perspectives. So, the detection accuracy combines the result in the competence of the CNN model to appropriately predict both *N* (negative) and *P* (positive) cases, and it is not potential to assess if the CNN model is better in accurately expecting *N* or *P*. Thus, a high accuracy value can be achieved by the excellent ability of the CNN model to predict only one of the possible classes accurately.

Therefore, when the performance accomplishment of a CNN model is assessed, other evaluation metrics of detection quality are also necessary to be estimated. Moreover, those evaluation metrics are very beneficial for comparing various CNN-based classification models because different CNN models can expect very distinct classes but with identical classification accuracy. Thus, different detection analysis metrics are exploited in this stage to explore the performance assessment of the developed nineteen CNN models for our investigated binary classification challenge of detecting ransomware attacks.

Our detection analysis of the binary classification challenge has been performed based on obtaining the results of the confusion matrix, loss and accuracy curves, validation accuracy, true-positive rate (TPR) (recall) (sensitivity), predictive value (PPV) (precision), negative predictive value (NPV), true-negative rate (TNR) (specificity), F1-Score, area under the receiver operating characteristic (AROC) curve, AROC score, false-negative rate (FNR), false-positive rate (FPR), false-omission rate (FOR), false-discovery rate (FDR), and misclassification rate.

**Figure 5:**
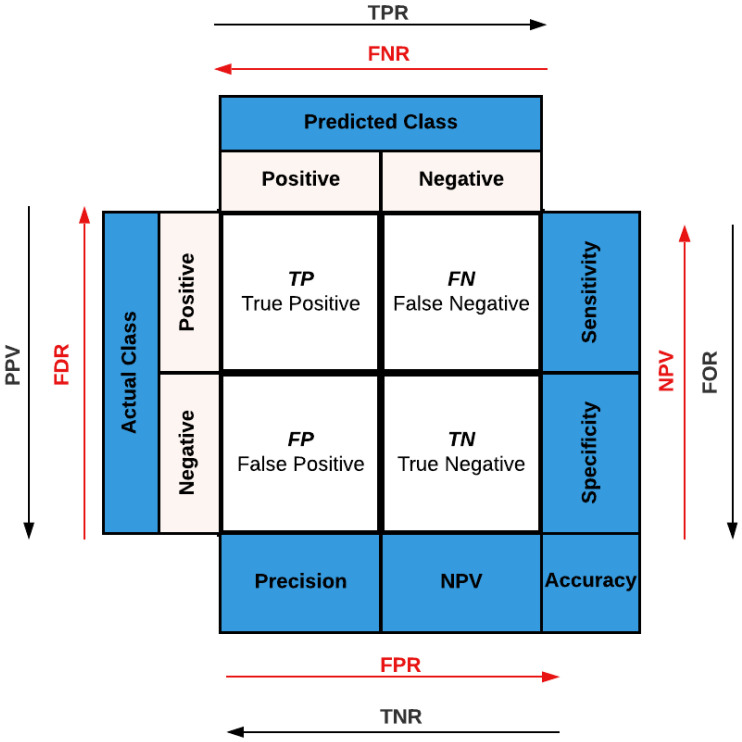
Binary confusion matrix.

In any binary classification challenge, if *N* (negative) and *P* (positive) are the probable classes/labels for each examination, a detection CNN model can generate only four scores, as shown in Figure 5. These scores are (1) *TP* (true positive): this score occurs if the CNN model predicts P, and it is likewise the true response, (2) *FP* (false positive): this score occurs if the CNN model predicts *P*, but the true response is *N*, (3) *TN* (true negative): this score occurs if the CNN model predicts *N*, and it is likewise the true response, and (4) *FN* (false negative): this score occurs if the CNN model predicts *N*, but the true response is *P*. These four scores can be utilized to estimate the numerical values of the examined detection assessment metrics; their mathematical formulas are expressed as follows:

(1) $$\;\mathrm{Validation}\;\mathrm{accuracy}\;=\frac{TN+TP}{FP+TP+FN+TN}$$

(2) $$\mathrm{TPR}\;\left(\mathrm{sensitivity}\right)\;=\;\mathrm{recall}=\frac{TP}{FN+TP}$$

(3) $$\mathrm{PPV}\;\left(\mathrm{precision}\right)=\frac{TP}{FP+TP}$$

(4) $$\mathrm{NPV}=\frac{TN}{FN+TN}$$

(5) $$\mathrm{TNR}\;\left(\mathrm{specificity}\right)=\frac{TN}{FP+TN}$$

(6) $$F1-\mathrm{Score}=\frac{2\mathrm{TP}}{2\mathrm{TP}+\mathrm{FN}+\mathrm{FP}}$$

(7) $$\mathrm{false}-\mathrm{negative}\;\mathrm{rate}\;\left(\mathrm{FNR}\right)=\frac{FN}{TP+FN}$$

(8) $$\mathrm{false}-\mathrm{positive}\;\mathrm{rate}\;\left(\mathrm{FPR}\right)=\frac{FP}{TN+FP}$$

(9) $$\mathrm{false}-\mathrm{omission}\;\mathrm{rate}\;\left(\mathrm{FOR}\right)=\frac{FN}{TN+FN}$$

(10) $$\mathrm{false}-\mathrm{discovery}\;\mathrm{rate}\;\left(\mathrm{FDR}\right)=\frac{FP}{TP+FP}$$

(11) $$\mathrm{misclassification}\;\mathrm{rate}=\frac{FN+FP}{FP+TP+FN+TN}$$

The loss curve is a tracing representation that demonstrates the estimated loss ratios for all examined training epochs, while the accuracy curve is a tracing representation that indicates the estimated accuracy ratios for all examined training epochs. Finally, the AROC curve represents the trade-off between the FPR (1-specificity) and TPR, while the AROC score is the estimated area under the AROC curve.

## 4. Results and Comparisons

This section introduces the analysis and discussions of the proposed E2E-RDS, including static-based and vision-based RD approaches using our collected and balanced benign and ransomware APKs. The dataset used in this work is publicly available on the Security Engineering Lab (SEL) website (https://sel.psu.edu.sa/Research/datasets/2020_RansIm-DS.php, accessed on 1 January 2020). The collection process of this dataset started by assembling the Android APKs (ransomware and benign). Both ransomware and benign samples were downloaded as Android Packages (APK). Benign APKs were downloaded from the Google play store. While ransomware APKs were downloaded from several famous market repositories, including HelDroid, VirusTotal, RansomProper, and Koodous. After that, the duplicated Android apps were removed.

### 4.1. Performance Analysis and Discussion of the Proposed Static-Based RD Approach

This section discusses the performance analysis of the proposed static-based RD approach. The evaluation of the utilized eight different ML classifiers is performed on the extracted features of the balanced ransomware and benign images (500 ransomware and 500 benign). These features are divided into 80% to train the layers of the examined ML classifiers and 20% for testing the utilized ML classifiers. These testing and training percentages of benign and ransomware features are selected randomly from the obtained static features dataset.

For simplicity in the presentation of the results, the obtained confusion matrix and ROC curve of only the best proficient ML classifier used in the proposed static-based RD approach are presented. At the same time, the summarized outcomes of the average values of all quantitive evaluation metrics (accuracy, recall, precision, and F1-Score) are offered for all examined ML classifiers.

**Figure 6:**
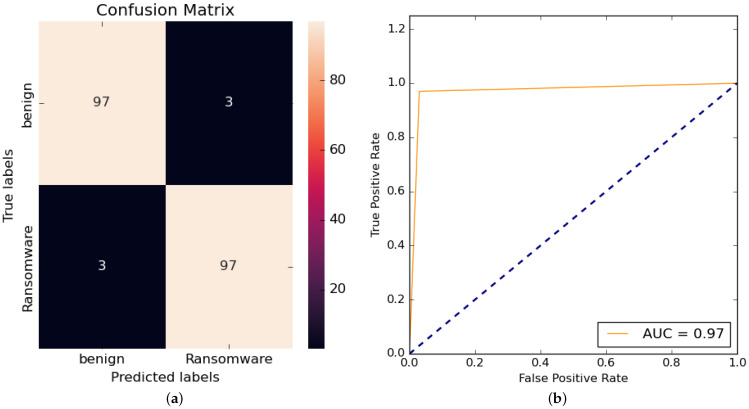
The obtained confusion matrix and ROC curve for the best accomplished ML-based AB detection model. (**a**) Confusion matrix. (**b**) ROC curve.

Figure 6 declares the obtained confusion matric and ROC curve for the best accomplished AB classification model used in the proposed static-based RD approach. The acquired confusion matrix and ROC curve reveal that the AB ML-based RD classifier can correctly detect almost all ransomware APKs and differentiate them from benign APKs using the static input features.

The performance analysis results of the average values of different classification assessment metrics for the examined ML classifiers are given in Table 5. The tested ML models utilized in the proposed static-based RD approach are observed to achieve reasonable and acceptable detection outcomes of high detection accuracy, F1-Score, recall, and precision values. The tested ML models utilized in the proposed static-based RD approach are observed to achieve reasonable and acceptable detection outcomes of high detection accuracy, F1-Score, recall, and precision values. The best-accomplished ML classifier that achieves high detection accuracy was the AB model, while the LR model achieved the lowest detection performance compared to other examined ML models.

### 4.2. Performance Analysis and Discussion of the Proposed Vision-Based RD Approach

In this section, the performance analysis of the proposed vision-based RD approach is discussed. The training and validation experiments were carried out using MATLAB 2020b on a laptop machine with an Intel Core i7-4500 processor with 8 gigabyte memory without a GPU accelerator. The evaluation of the 19 different employed CNN models is performed on the created balanced visual ransomware and benign images (500 ransomware images and 500 benign images). These images were divided into 80% to train the layers of the examined CNN models and 20% for testing the utilized CNN models. These testing and training percentages of benign and ransomware images were selected randomly from the created visual color or gray dataset.

Therefore, the detection efficiency and classification performance of the proposed vision-based RD approach that composes nineteen different employed CNN models have been examined using both visual color and grayscale images generated from our collected ransomware and benign apps. This is to extensively analyze and investigate the effect of using two different visual image representations (color and grayscale formats) of the android ransomware and benign APKs on the detection efficiency of the proposed vision-based RD approach.

For simplicity in the presentation of the results, the detailed outcomes regarding the confusion matrix, loss and accuracy curves, and AROC curves of the best proficient fine-tuned ResNet50 model in the proposed vision-based RD approach are presented. While the summarized outcomes of the average values of all quantitive evaluation metrics (validation accuracy (Val. Acc.), recall (Rec.), precision (Prec.), NPV, specificity (Spec.), F1-Score, AROC score, FNR, FPR, FOR, FDR, and misclassification rate (Mis. Class. Rate)) that are discussed in Section 3.2.3 are offered for all developed and fine-tuned CNN models utilized in the proposed vision-based RD approach.

**Figure 7:**
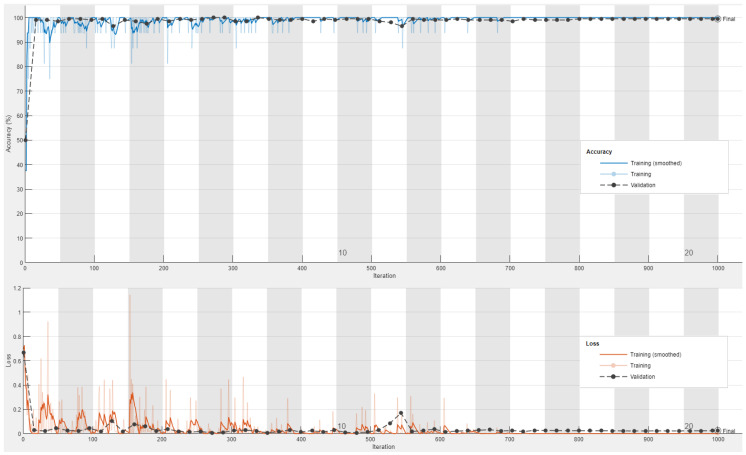
The obtained accuracy and loss curves for the training and validation processes of the best accomplished ResNet50 classification model using the color image dataset.

**Figure 8:**
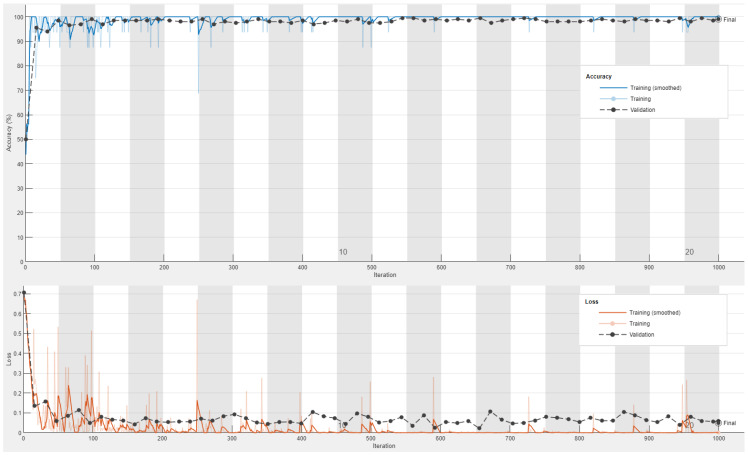
The obtained accuracy and loss curves for the training and validation processes of the best accomplished ResNet50 classification model using the gray image dataset.

Figure 7 and Figure 8 declare the obtained accuracy and loss curves for the training and validation processes of the best accomplished ResNet50 classification model using the color and grayscale image datasets, respectively.

It can be realized that the testing and training accuracy curves for both color and grayscale datasets are matched to each other, and they tend to be steady after only five epochs. However, the testing loss curves were marginally higher than the training loss curves, especially for the grayscale image dataset, but the obtained validation loss has an acceptable average value of less than 0.1. Thus, it is demonstrated that the outcomes of the testing and training loss and accuracy curves for the visual color dataset are better than those for the visual grayscale dataset. Both visual color and grayscale datasets proved and validated the excellent detection performance of the examined fine-tuned ResNet50 model using different visual image representations for the ransomware and benign APKs. So, the fine-tuned ResNet50 model achieved high detection and low misclassification performance at minimal epochs (training iterations). Furthermore, analogous outstanding achievements of loss and accuracy curves are acquired and accomplished for the other investigated 18 different CNN models tested in the proposed vision-based RD approach for the color and grayscale image datasets. Consequently, the developed fine-tuned CNN models that have been used in the proposed vision-based RD approach are highly recommended to be exploited in cybersecurity applications for accurately detecting ransomware attacks in Android operating systems.

**Figure 9:**
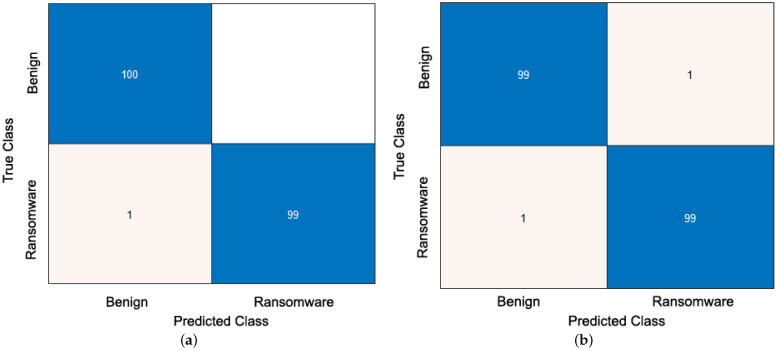
The obtained confusion matrix for the best accomplished ResNet50 classification model using (**a**) color image dataset and (**b**) gray image dataset.

**Figure 10:**
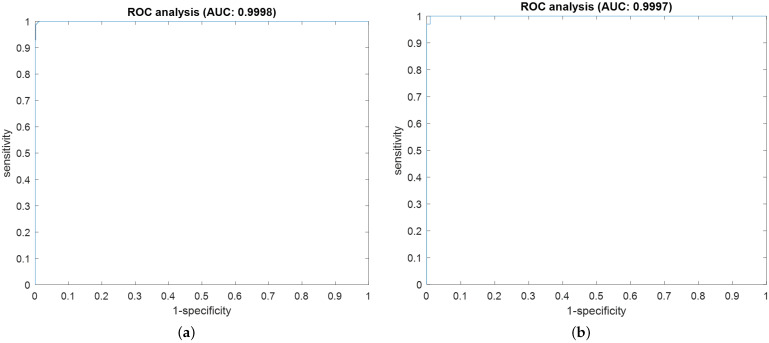
The obtained AROC curves for the best accomplished ResNet50 classification model using (**a**) color image dataset and (**b**) gray image dataset.

Figure 9 shows the obtained confusion matrices for the best accomplished FT ResNet50 classification model using color and grayscale image datasets. In addition, Figure 10 demonstrates the obtained AROC curves for the best accomplished ResNet50 classification model using color and grayscale image datasets. These acquired confusion matrices and AROC curves reveal that the fine-tuned ResNet50 model can correctly detect almost all ransomware APKs and differentiate them from benign APKs using color or grayscale image datasets. Furthermore, it is clear that the attained confusion matrices or AROC curves of the color image dataset are slightly better than those achieved using the grayscale image dataset. These similar observations are obtained for the other 18 different examined FT CNN models utilized in the proposed vision-based RD approach. Thus, the employed fine-tuned CNN models are highly recommended to be used efficiently for detecting ransomware attacks in Android cybersecurity applications.

The performance analysis results of the average values of different classification assessment metrics for the examined CNN models on the color and grayscale image datasets are given in Table 6 and Table 7, respectively. It is observed that the whole tested fine-tuned CNN models utilized in the proposed vision-based RD approach achieve promising outcomes of high validation accuracy, TPR, PPV, NPV, TNR, F1-Score, and AROC values, and also, all of them attain low FNR, FPR, FOR, FDR, and misclassification rate values for both color and grayscale datasets. Moreover, it is demonstrated that the assessment detection values obtained using the color image dataset are slightly higher than those obtained using the grayscale image dataset. Furthermore, despite its design simplicity, the fine-tuned ResNet50 CNN model achieved the best detection results compared to other fine-tuned CNN models for both color and grayscale images. These promising classification results for the whole tested FT CNN-based TL models have been accomplished with the aid of using transfer learned features resulting from the pre-trained CNN models in addition to exploiting the fine-tuning of their layers and hyperparameter values.

After validating the classification performance analysis and detection efficiency of the proposed fine-tuned CNN models, we have investigated the complexity analysis of the proposed vision-based RD approach concerning (1) the storage size of the utilized visual datasets, (2) experimental requirements of the employed CNN models, and (3) computation detection overhead of the FT CNN models. Table 8 presents the storage size in gigabytes of the color and grayscale visual images of the ransomware and benign samples, while Table 9 indicates the specifications of the utilized FT CNN models. Finally, Table 10 introduces the computational analysis of the utilized FT CNN models.

Table 8 shows that the storage size of the visual color samples (4.198 GB) has a minor increase in size compared to the storage size of the visual grayscale samples (4.143 GB). The last row in Table 8 indicates that there is a slight storage size increase of 0.055 GB for the visual color samples compared to the visual grayscale samples. Thus, there is an increase in percentage of 1.31% produced when using the visual color samples, which is reasonable and tolerable compared to the added benefits from using them compared to visual grayscale samples concerning the accomplished high detection efficiency and low misclassification rate for almost all FT CNN models as discussed and indicated previously in Table 6 and Table 7.

The disk size, total number of layers, number of parameters (total, trainable, and non-trainable), and reduction percentage in training parameters of the whole employed FT CNN models in the proposed vision-based RD approach are stated in Table 9. As indicated, these experimental requirements and specifications differ from one CNN model to another CNN model, depending on the model structure (single-path design or multi-path design) and the total number of included CNN layers. Furthermore, the number of training and non-training parameters of each CNN model is based on the number of frozen and unfrozen layers. In the proposed vision-based RD approach, most of the training parameters of the CNN layers of the utilized fine-tuned models are frozen. So, for example, the FT ResNet50 model has trainable parameters of 4,096 and non-trainable parameters of 25,595,904, and thus there is a high reduction percentage in training parameters reached 99.98%. This significant reduction in training parameters comes from the valuable utilization of transfer learned features resulting from the pre-trained CNN models and exploiting the considerable advantages of fine-tuning the hyperparameters, weights, and CNN layers of the pre-trained CNN-based TL models as indicated in Table 9, the same observations concerning the number of training parameters and their reduction percentage are obtained for all examined FT CNN models in the proposed vision-based RD approach.

The comprehensive computational analysis of the FT CNN models for the visual color and grayscale samples are introduced in Table 10. The total training and validation time and the average detection time to identify the ransomware or benign sample for each CNN model are estimated. As observed, the processing time is different from one CNN model to another CNN model, depending on the number of training parameters and CNN layers as clarified in Table 9. It can be concluded that the computational analysis of the whole tested FT CNN models proves that the average validation time spent to recognize ransomware samples or benign samples is acceptable for all FT CNN models, comparable to their accomplished classification accuracy.

The overall extensive validation of the proposed E2E-RDS has proven its efficiency in detecting ransomware, whether by reverse engineering the code itself and then analyzing it or by handling it in its current format and applying vision-based analysis. The intelligence is applied using ML or CNN models while considering the system’s resources and requirements. Finally, the main target is achieved in detecting ransomware with high accuracy to protect users’ machines and data.

In addition to the presented results, we also compared the proposed RDS system with other related works that have been evaluated on the same dataset. Table 11 shows the comparative analysis outcomes. Compared to related works evaluated on the same dataset, it is noticed that the proposed RDS system offers a high detection performance for both static-based ML and vision-based DL approaches. So, compared to the other systems, the achieved detection accuracy of the proposed system indicates its superiority in detecting ransomware.

## 5. Conclusions and Future Work

Ransomware still registers high numbers of successful attacks with a significant impact. Therefore, many researchers are still working to enhance the performance of ransomware detection, especially in the context of Android systems. However, the existing approaches usually utilize one of the analytical approaches that either reverse engineer the ransomware or deal with it in its current format. Both approaches were used in the literature but (a) separately by applying one of them only and (b) differently by considering various contexts, datasets, simulation environments, and evaluation metrics. This paper comprehensively analyses Android ransomware by introducing an efficient end-to-end ransomware detection system (E2E-RDS). The proposed E2E-RDS either reverses engineering the ransomware code, parsing it, extracting its essential features, and then building predictive systems that are based on machine learning (ML) classifiers, or keeps the ransomware in its executable format, converts it to images and then build predictive systems using vision-based CNN models.

The proposed E2E-RDS aims to assess the capabilities of applying different analysis models while considering the same context in terms of ransomware datasets, experimental environments, and evaluation metrics. The purpose is to highly and efficiently recognize the ransomware apps and prevent the users from installing them while considering the system resources and requirements. The experiments reveal that the resulting predictive models from different analysis systems succeeded in reaching 99.5% detection accuracy. Eight ML classifiers and 19 CNN models were developed and examined to reach this accuracy. Moreover, extensive experiments were conducted to examine not only the accuracy but many other metrics related to security and complexity. The target is to highlight all aspects related to malware detection in general and ransomware in specific to choose the proper model while building the ransomware detection systems.

In future work, different malware types other than ransomware could be investigated. Additionally, different ML and CNN models could also be integrated and examined within the E2E-RDS. Moreover, we intend to build an end-to-end mathematical analysis for the proposed RDS system. Furthermore, we target to test the detection performance of the proposed RDS system on real-world applications and services.

## Figures and Tables

**Table 2:** Typical image widths for different sizes of benign and ransomware APKs.

APK Size	Image Width
<10 KB	32
10∼30 KB	64
30∼60 KB	128
60∼100 KB	256
100∼200 KB	384
200∼500 KB	512
500∼1000 KB	768
1000∼2000 KB	1024
2000∼4000 KB	1280
4000∼8000 KB	1536
8000 KB∼10 MB	1792
10∼15 MB	2048
15 ∼20 MB	2560
20∼25 MB	3072
25∼30 MB	4096
>30 MB	5120

**Table 3:** The examined CNN models and their input image resolutions.

No.	Model Name	Image Size
1	ResNet50	224 × 224
2	AlexNet	227 × 227
3	InceptionV3	299 × 299
4	ResNet101	224 × 224
5	GoogleNet	224 × 224
6	VGG16	224 × 224
7	DarkNet53	256 × 256
8	Xception	299 × 299
9	InceptionResNetV2	299 ×299
10	MobileNetV2	224 × 224
11	NasNetMobile	224 × 224
12	DarkNet19	256 × 256
13	ResNet18	224 × 224
14	DenseNet201	224 × 224
15	NasNetLarge	331 × 331
16	Places365-GoogleNet	224 × 224
17	ShuffleNet	224 × 224
18	SqueezeNet	227 × 227
19	VGG19	224 × 224

**Table 4:** Fine-tuning and training optimization parameters utilized in the proposed vision-based RD approach.

Parameter	Value
Learning ratio (LR)	0.00001
Optimization approach	ADAM
Regularization approach	L2-regularizer
Regularization decay rate	0.001
Number of epochs	20
Minimum batch size	16
Validation frequency	16
Dropout rate	0.5
LR schedule parameter	Piecewise
LR drop period parameter	3
LR drop factor parameter	0.9
Loss function	Categorical cross-entropy
Shuffling scenario	Performed every epoch

**Table 5:** The obtained evaluation metrics for the examined ML detection models.

ML Model	Accuracy	Recall	Precision	F1-Score
SVM	0.925	0.925	0.925	0.925
RF	0.945	0.945	0.945	0.94
AB	0.97	0.97	0.97	0.97
CART	0.94	0.94	0.94	0.94
KNN	0.93	0.929	0.93	0.93
NB	0.88	0.88	0.889	0.88
LDA	0.90	0.90	0.90	0.90
LR	0.84	0.84	0.84	0.84

**Table 6:** Performance analysis results of the examined CNN models on color image dataset.

Model	Val. Acc.	Rec. (TPR)	Prec. (PPV)	NPV	Spec. (TNR)	F1-Score	AROC	FNR	FPR	FOR	FDR	Mis Class. Rate
**ResNet50**	0.995	1	0.99	1	0.99	0.995	0.9998	0	0.01	0	0.0099	0.005
**AlexNet**	0.995	0.99	1	0.9901	1	0.995	1	0.01	0	0.0099	0	0.005
**InceptionV3**	0.995	0.99	1	0.9901	1	0.995	0.999	0.01	0	0.0099	0	0.005
**ResNet101**	0.995	0.99	1	0.9901	1	0.995	0.9998	0.01	0	0.0099	0	0.005
**GoogleNet**	0.99	0.99	0.99	0.99	0.99	0.99	0.9977	0.01	0.01	0.01	0.01	0.01
**VGG16**	0.99	0.98	1	0.9804	1	0.9899	1	0.02	0	0.0196	0	0.01
**DarkNet53**	0.99	0.98	1	0.9804	1	0.9899	0.99	0.02	0	0.0196	0	0.01
**Xception**	0.99	0.99	0.99	0.99	0.99	0.99	0.9981	0.01	0.01	0.01	0.01	0.01
**InceptionResNetV2**	0.99	1	0.9804	1	0.98	0.99	0.9981	0	0.02	0	0.0196	0.01
**MobileNetV2**	0.985	1	0.9709	1	0.97	0.9852	0.9995	0	0.03	0	0.0291	0.015
**NasNetMobile**	0.985	0.99	0.9802	0.9899	0.98	0.985	0.9984	0.01	0.02	0.0101	0.0198	0.015
**DarkNet19**	0.985	0.99	0.9802	0.9899	0.98	0.985	0.9926	0.01	0.02	0.0101	0.0198	0.015
**ResNet18**	0.985	1	0.9708	1	0.97	0.9852	0.9962	0	0.03	0	0.0291	0.015
**DenseNet201**	0.985	1	0.9708	1	0.97	0.9852	0.9988	0	0.03	0	0.0291	0.015
**NasNetLarge**	0.985	0.99	0.9802	0.9899	0.98	0.985	0.9984	0.01	0.02	0.0101	0.0198	0.015
**Places365-GoogleNet**	0.98	1	0.9615	1	0.96	0.9804	0.9991	0	0.04	0	0.0384	0.02
**ShuffleNet**	0.98	0.97	0.9898	0.9706	0.99	0.9798	0.9904	0.03	0.01	0.0294	0.0102	0.02
**SqueezeNet**	0.98	0.99	0.9706	0.9897	0.97	0.9802	0.9973	0.01	0.03	0.0102	0.0294	0.02
**VGG19**	0.97	0.97	0.97	0.97	0.97	0.97	0.995	0.03	0.03	0.03	0.03	0.03

**Table 7:** Performance analysis results of the examined CNN models on gray image dataset.

Model	Val. Acc.	Rec. (TPR)	Prec. (PPV)	NPV	Spec. (TNR)	F1-Score	AROC	FNR	FPR	FOR	FDR	Mis Class. Rate
**ResNet50**	0.99	0.99	0.99	1	0.99	0.99	0.9997	0.01	0.01	0.01	0.01	0.01
**NasNetMobile**	0.985	0.99	0.9802	0.9899	0.98	0.9851	0.9983	0.01	0.02	0.0101	0.0198	0.015
**MobileNetV2**	0.985	0.97	1	0.9709	1	0.9847	0.9912	0.03	0	0.0291	0	0.015
**InceptionV3**	0.985	0.97	1	0.9709	1	0.9847	0.9999	0.03	0	0.0291	0	0.015
**GoogleNet**	0.985	0.98	0.9899	0.9802	0.99	0.9849	0.9997	0.02	0.01	0.0198	0.0101	0.015
**VGG16**	0.98	0.96	1	0.9615	1	0.9796	0.9996	0.04	0	0.0385	0	0.02
**AlexNet**	0.98	0.98	0.98	0.98	0.98	0.98	0.9939	0.02	0.02	0.02	0.02	0.02
**DarkNet19**	0.98	0.98	0.98	0.98	0.98	0.98	0.9925	0.02	0.02	0.02	0.02	0.02
**Places365-GoogleNet**	0.975	1	0.9523	1	0.95	0.9756	0.9942	0	0.05	0	0.0476	0.025
**ResNet18**	0.975	1	0.9523	1	0.95	0.9756	0.9996	0	0.05	0	0.0476	0.025
**ResNet101**	0.975	0.97	0.9798	0.9703	0.98	0.9749	0.9949	0.03	0.02	0.0297	0.0202	0.025
**DarkNet53**	0.975	0.98	0.9703	0.9798	0.97	0.9751	0.999	0.02	0.03	0.0202	0.0297	0.025
**ShuffleNet**	0.975	0.97	0.9798	0.9703	0.98	0.9749	0.9986	0.03	0.02	0.0297	0.0202	0.025
**Xception**	0.975	0.96	0.9897	0.9612	0.99	0.9746	0.9992	0.04	0.01	0.0388	0.0103	0.025
**InceptionResNetV2**	0.975	0.99	0.9612	0.9897	0.96	0.9754	0.9985	0.01	0.04	0.0103	0.0388	0.025
**SqueezeNet**	0.97	0.96	0.9796	0.9608	0.98	0.9696	0.9871	0.04	0.02	0.0392	0.0204	0.03
**DenseNet201**	0.97	0.96	0.9796	0.9608	0.98	0.9696	0.998	0.04	0.02	0.0392	0.0204	0.03
**VGG19**	0.96	0.95	0.9694	0.9509	0.97	0.9596	0.9962	0.05	0.03	0.049	0.0306	0.04
**NasNetLarge**	0.96	0.99	0.934	0.9894	0.93	0.9612	0.9943	0.01	0.07	0.0106	0.066	0.04

**Table 8:** The storage size (gigabytes) of the color and gray images of the ransomware and benign samples.

Android Apps	Color Samples	Gray Samples
Ransomware	0.398	0.353
Benign	3.8	3.79
Total	4.198	4.143
Additional storage size/incremental percentage produced by color samples	0.055 GB/1.31%	

**Table 9:** Specifications of the utilized FT CNN models.

CNN Model	Disk Size (MB)	Layers	Parameters (Total)	Parameters (Trained)	Parameters (Non-Trained)	Reduced (%) in Training Parameters
**ResNet50**	96	50	25,600,000	4096	25,595,904	99.98
**AlexNet**	227	8	61,000,000	8192	60,991,808	99.99
**InceptionV3**	89	48	23,900,000	4096	23,895,904	99.98
**ResNet101**	167	101	44,600,000	4096	44,595,904	99.99
**GoogleNet**	27	22	7,000,000	2048	6,997,952	99.97
**VGG16**	515	16	138,000,000	8192	137,991,808	99.99
**DarkNet53**	155	53	41,600,000	2048	41,597,952	99.99
**Xception**	85	71	22,900,000	4096	22,895,904	99.98
**InceptionResNetV2**	209	164	55,900.00	3072	55,896,928	99.99
**MobileNetV2**	13	53	3,500,000	2560	3,497,440	99.93
**NasNetMobile**	20	*	5,300,000	2112	5,297,888	99.96
**DarkNet19**	78	19	20,800,000	2048	20,797,952	99.99
**ResNet18**	44	18	11,700,000	1024	11,698,976	99.99
**DenseNet201**	77	201	20,000,000	3840	19,996,160	99.98
**NasNetLarge**	332	*	88,900,000	8064	88,891,936	99.99
**Places365-GoogleNet**	27	22	61,000,000	2048	60,997,952	99.99
**ShuffleNet**	5.4	50	7,000,000	1024	6,998,976	99.99
**SqueezeNet**	5.2	18	1,240,000	1024	1,238,976	99.92
**VGG19**	535	19	144,000,000	8192	143,991,808	99.99

* The NasNetLarge and NasNetMobile models do not comprise a linear structure of CNN modules.

**Table 10:** Computational analysis of the utilized FT CNN models.

CNN Model	Color Samples		Gray Samples	
	**Total Execution Time (s)**	**Average Time per Sample (s)**	**Total Execution Time (s)**	**Average Time per Sample (s)**
**ResNet50**	1617	1.62	1499	1.5
**AlexNet**	1890	1.89	1714	1.71
**InceptionV3**	2166	2.17	2050	2.05
**ResNet101**	2183	2.18	1939	1.94
**GoogleNet**	1478	1.48	1434	1.43
**VGG16**	1966	1.97	1803	1.8
**DarkNet53**	1975	1.98	1877	1.88
**Xception**	2021	2.02	1934	1.93
**InceptionResNetV2**	3792	3.79	3712	3.71
**MobileNetV2**	1636	1.64	1604	1.6
**NasNetMobile**	4793	4.79	4609	4.61
**DarkNet19**	1400	1.4	1377	1.38
**ResNet18**	1377	1.38	1279	1.28
**DenseNet201**	4683	4.68	4623	4.62
**NasNetLarge**	5059	5.06	4967	4.97
**Places365-GoogleNet**	1496	1.5	1410	1.41
**ShuffleNet**	1569	1.57	1555	1.56
**SqueezeNet**	1313	1.31	1248	1.25
**VGG19**	2220	2.22	2147	2.15

**Table 11:** Comparative analysis outcomes.

Detection System	Detection Accuracy
Proposed (FT ResNet5)	99.5
Proposed (Ada Boost)	97
[1]	94.5
[2]	96.4
[58]	64.8
[59]	96.2

## Data Availability

Not applicable.
